# Outcome of intensive care treatment for lung cancer patients

**DOI:** 10.1186/cc12407

**Published:** 2013-03-19

**Authors:** L Hajjar, F Galas, D Goto, A Tavares, M Santos, A Freitas, T Oliveira, P Tonini, L Cavichio, M Bazan, J Almeida, M Cavalcante, J Fukushima, S Vieira, E Osawa

**Affiliations:** 1 Heart Institute, São Paulo, Brazil; 2 Cancer Institute of University of São Paulo, Brazil

## Introduction

Patients with lung cancer commonly require the ICU for a variety of acute illnesses related to the underlying malignancy, treatment, or comorbid conditions. ICU admission of patients with nonresectable lung cancer has been criticized based on the high mortality rate in this population. However, recent advances in critical care may have changed this scenario. The aim of this study was to identify factors associated with hospital mortality in this group of patients.

## Methods

A retrospective study was conducted in consecutive medical and surgical patients with lung cancer admitted to a university hospital ICU in São Paulo, between 2007 and 2012. A univariate analysis was performed to identify associated variables with hospital mortality. Selected variables were included in the multivariate model.

## Results

From 597 patients included in the study, 477 were medical admissions (79.8%) and 120 were surgical admissions (20.2%). Four hundred and twenty (70%) patients had metastasis, 222 patients (37%) required the ICU because of respiratory failure and 121 (20%) because of septic shock. The ICU and hospital mortality rates were 53.9% and 64%, respectively. In the univariate analysis, variables associated with hospital mortality were diagnosis of nonsmall-cell lung cancer, higher Charlson morbidity index, medical admission, active neoplasm, vasopressor need at admission to and at 24 hours of ICU, acute renal failure at admission, non-invasive ventilation or mechanical ventilation need at admission to and at 24 hours of ICU and a higher admission arterial lactate. By multivariate analysis, risk factors of hospital mortality were diagnosis of nonsmall-cell lung cancer (OR = 3.32; 95% CI, 2.08 to 5.29, *P *0.001), medical admission (OR = 2.89; 95% CI, 1.75 to 4.77, *P *0.001), acute renal failure at admission (OR = 4.09; 95% CI, 1.49 to 11.21, *P *0.001), non-invasive ventilation at 24 hours of ICU (OR = 2.50; 95% CI, 1.48 to 4.23, *P *= 0.001) and mechanical ventilation at 24 hours of ICU (OR = 3.68; 95% CI, 1.87 to 7.26, *P *0.001).

## Conclusion

Hospital survival in patients with lung cancer requiring ICU admission was 36%. Our results provide evidence that ICU management may be appropriate in patients with nonresectable lung cancer and appoint risk factors for mortality, helping to better triage cancer patients who will benefit from ICU care.

